# Developing of Low-Cost Air Pollution Sensor—Measurements with the Unmanned Aerial Vehicles in Poland

**DOI:** 10.3390/s20123582

**Published:** 2020-06-24

**Authors:** Sławomir Pochwała, Arkadiusz Gardecki, Piotr Lewandowski, Viola Somogyi, Stanisław Anweiler

**Affiliations:** 1 Department of Mechanical Engineering, Opole University of Technology, 45-271 Opole, Poland; pi.lewandowski@student.po.edu.pl (P.L.); s.anweiler@po.edu.pl (S.A.); 2 Department of Automatic Control and Informatics, Opole University of Technology, 45-758 Opole, Poland; a.gardecki@po.edu.pl; 3 Faculty of Engineering, University of Pannonia, 8200 Veszprem, Hungary; somogyiv@uni-pannon.hu

**Keywords:** air pollution, sensor, UAV, particulate matter, vertical profile

## Abstract

This article presents the capabilities and selected measurement results from the newly developed low-cost air pollution measurement system mounted on an unmanned aerial vehicle (UAV). The system is designed and manufactured by the authors and is intended to facilitate, accelerate, and ensure the safety of operators when measuring air pollutants. It allows the creation of three-dimensional models and measurement visualizations, thanks to which it is possible to observe the location of leakage of substances and the direction of air pollution spread by various types of substances. Based on these models, it is possible to create area audits and strategies for the elimination of pollution sources. Thanks to the usage of a multi-socket microprocessor system, the combination of nine different air quality sensors can be installed in a very small device. The possibility of simultaneously measuring several different substances has been achieved at a very low cost for building the sensor unit: 70 EUR. The very small size of this device makes it easy and safe to mount it on a small drone (UAV). Because of this device, many harmful chemical compounds such as ammonia, hexane, benzene, carbon monoxide, and carbon dioxide, as well as flammable substances such as hydrogen and methane, can be detected. Additionally, a very important function is the ability to perform measurements of PM_2.5_ and PM_10_ suspended particulates. Thanks to the use of UAV, the measurement is carried out remotely by the operator, which allows us to avoid the direct exposure of humans to harmful factors. A big advantage is the quick measurement of large spaces, at different heights above the ground, in different weather conditions. Because of the three-dimensional positioning from GPS receiver, users can plot points and use colors reflecting a concentration of measured features to better visualize the air pollution. A human-friendly data output can be used to determine the mostly hazardous regions of the sampled area.

## 1. Introduction

The Earth itself introduces a certain amount of pollution into the atmosphere as a result of various natural phenomena, but human activities are adding to this pollution by releasing more dangerous tiny particles (aerosols) and gases into the Earth’s atmosphere [1]. The environmental impact of the energy sector, the security, and the economics of energy supply and utilization have been raising amassed concerns [2]. The growing global consumption of energy and goods accelerates the depletion of non-renewable resources and increases environmental pollution [3]. Sustainable development increasingly requires an integrated approach, especially in the context of addressing the climate crisis [4]. The pursuit of sustainable development gradually introduces more and more problems related to climate change [5,6]. Air pollution caused by energy generation and consumption is both a global and local issue. It contributes to global warming along with degradation in human health, ecosystem health, and local and global sustainable development [7]. Its effect is amplified by terrain shape and profile. This is a vital issue, especially for industrialized urban conglomerates [8]. The meteorological and atmospheric factors contributing to the problems have been reported widely [9]. Cities are acknowledged as being responsible for the majority of global primary energy use, which has accounted for above 70% of CO_2_ global emissions [10]. This applies to both the outdoor and the indoor environments [11]. These problems affect the whole world, but are still particularly important in the economies of individual countries [12,13]. Particulate and gaseous matter have a serious impact on human health [14,15,16]. There is a large variety of models concerning atmospheric pollution used, starting from computational fluid dynamics (CFD) models on a small scale, such as a street canyon [17,18], and spanning the geographic scales to regional and hemispheric models for the pollutants with enough lifetime in the atmosphere [19]. Some of the techniques like Eulerian modeling [20] or CFD modeling [21] are considering spatially three-dimensional processes, with a sophisticated description of the processes alongside each dimension [22].

The use of unmanned aerial vehicles (UAVs) for measurements for investigating the horizontal and vertical distribution of gaseous and particulate air pollutants in the atmospheric boundary layer (ABL) [23] is widely accepted by different types of UAVs. Airplanes [24,25], multicopters [26,27,28], and balloons are used in different locations all over the world, among others in Asia [29], and Europe [30]. Diversity in UAV use also applies to the measurement of different components of the atmosphere, for example, quadrotor for gaseous [31] and aerosol [25], and hexacopter for the airflow field in a hover state [32,33].

Although numerical simulations allow for convenient approximations of the phenomena occurring in ABL [34], the use of drones now makes it possible to verify and expand research in many other areas from agriculture to energy [35,36,37,38,39,40,41,42,43]. Drones allow for high-resolution measurements, the measurements of vertical profiles of PM_2.5_ concerning air humidity [44], measurements on the impact of aerosols on surface ozone concentration [19], and the spatial distribution of aerosols in specific location and time [45,46], like mountain valleys [47], rural riverside areas [48], and temperature [49]. Increasingly, attention is being paid to interdependencies and correlations between the different components of the atmosphere [50,51] and various events during air pollution episodes [52,53]. Unmanned aerial vehicles (UAVs) can also support preliminary visual inspections of boilers in power production blocks. Drones, properly selected and constructed, can support the processes of maintaining and managing heating and electricity networks [54,55,56]. The drones are also used during monitoring and cyclical measurement of the volume of stored or accumulated goods, or orthophoto mapping [57]. In production units, drones are ideally suited for assessing the technical condition of chimneys and cooling towers [58,59]. 

Therefore, developing low-cost air pollution multisensory arrays and measurements of the atmosphere condition with the application of unmanned aerial vehicles (UAV) is a forward-looking way to learn about and then reduce the negative impact of human activity on the quality of the atmosphere. 

The paper presents results and conclusions from the application of the developed test system for measuring air pollution based on a multisensory array mounted on an unmanned aerial vehicle (UAV). Thanks to the usage of a multi-socket microprocessor system, it is possible to measure up to nine different air pollutants simultaneously. The wireless connection enables us to save data locally and remotely. Data evaluation is carried out by a flying unit carrying a device designed and manufactured by the authors, which is the main focus of the article.

The system is designed to facilitate and accelerate the measurement of air pollution, and ensure the safety of its operators. It allows us to create three-dimensional measurement models, which then can be used to determine the location of substance leaks and the direction of air pollution propagation. Area audits and strategies for repairing pollution sources can be created based on these models. Due to using different combinations of air sensors mounted on a UAV (drone), many harmful compounds can be detected: ammonia, hexane, benzene, carbon monoxide, and carbon dioxide, as well as flammable substances: hydrogen, methane, natural gas, LP gas, and suspended dust—particulate matter PM_2.5_ and PM_10_.

By using a drone, the measurement is carried out remotely by the operator, avoiding direct human exposure to harmful factors. The major advantage is the quick scouring of the large surface area on various height levels above the ground.

## 2. Materials and Methods

**Figure 1:**
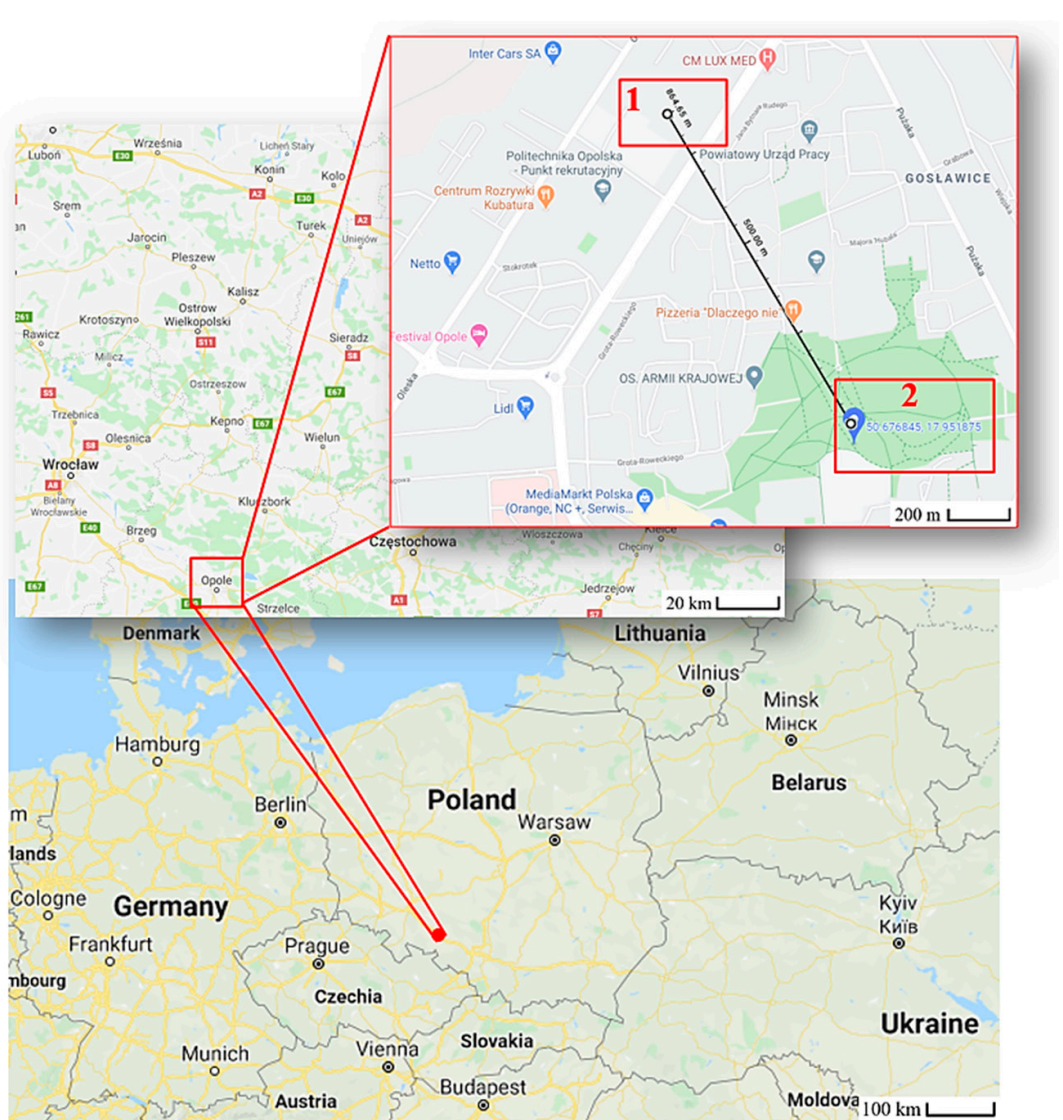
Location number 1 and 2 of the places where air pollutants were measured with the low-cost multisensory device mounted on an unmanned aerial vehicle (UAV).

The research regarding air pollution measurements with the use of a UAV was planned and carried out in the two places inside the urbanized area in the city of Opole (Poland). The location is shown in Figure 1. Location number 1 is at the Opole University of Technology near a busy road (100 m). Location number 2 is in a large urban recreational park at a considerable distance from busy roads (500 m).

**Figure 2:**
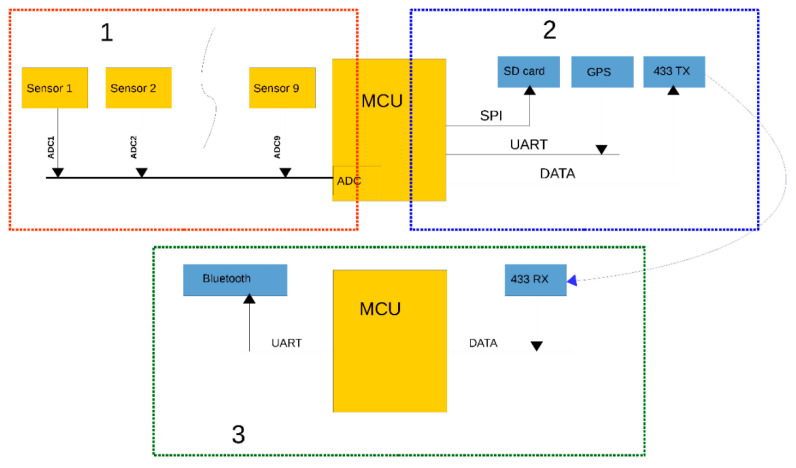
Measurement system diagram measuring (1) the sensor module, (2) the data module, and (3) the receiver module.

The air quality measuring system needed to meet some objectives for use on a UAV (drone) such as low weight and non-interfering wireless transmission. Figure 2 shows a simplified schematic of the device, and it is divided into three parts: measuring module (1), data module (2), and receiver module (3). The first two of which are mounted on the drone, and the last one allows us to receive live data; it stays with the UAV operator.

To acquire the samples, nine semiconductor sensors from the MQ series (for methane, hydrogen, and hexene measurements) [60], Figaro TGS (for benzene measurements) [61], and PMS7003 (for PM_2.5_, PM_10_ measurements) [62] have been used. Table 1 presents detailed information on the sensors used in the low-cost multisensory array preparation. 

MQ sensors series are produced in semiconductor technology. They are relatively inexpensive, as opposed to other types of sensors. They have fast response time and high sensitivity, and they are also excellent in long-life applications. 

According to the sensor datasheet, the measurement error for values below 100 μg/m^3^ is a constant value of 10 μg/m^3^, and for measured values above 100 μg/m^3^, it is 10% of the measured value. The resolution of the PM sensor (Plantower model 7003) is 1 μg/m^3^. The single response time is 1 s.

**Figure 3:**
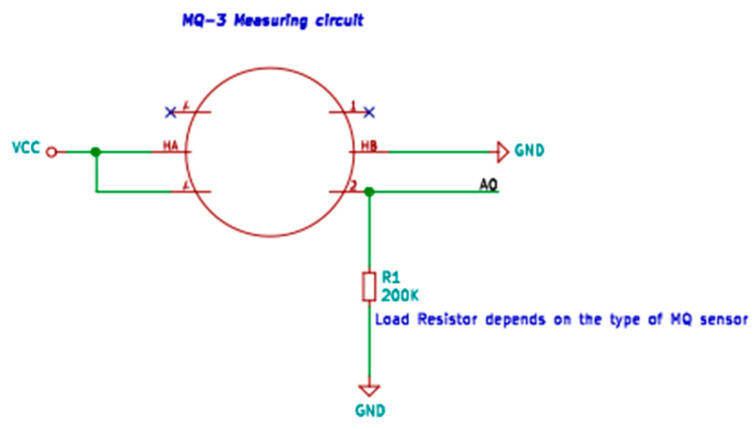
Single sensor circuit diagram.

Sensing the element resistance depends on the concentration of the target gas, and while using a sensor in the system of the voltage divider, the output voltage is logarithmically dependent on the concentration of applied gas. Using analog to digital converter (ADC) and our software, we can read and covert voltage to actual concentration in ppm. According to the datasheets, the maximum power of the sensors is 800 mW each. Using the operating voltages 5 V, the maximum current draw equals 160 mA. 10 sensors draw 1.44 A. When all sensors are turned on, it could run stable up to 1 h on a typical 7400 mWh power source. When the device is measuring only particulate matter (PM) concentration, it could work for 12 h. Sensors are connected with onboard voltage dividers that allow us to read the voltage using a 10-bit analog-digital converter, which comes as a built-in ATmega 328 microcontroller unit (MCU) [63]. The sample schematic diagram of the MQSeries sensor circuit is shown in Figure 3.

**Figure 4:**
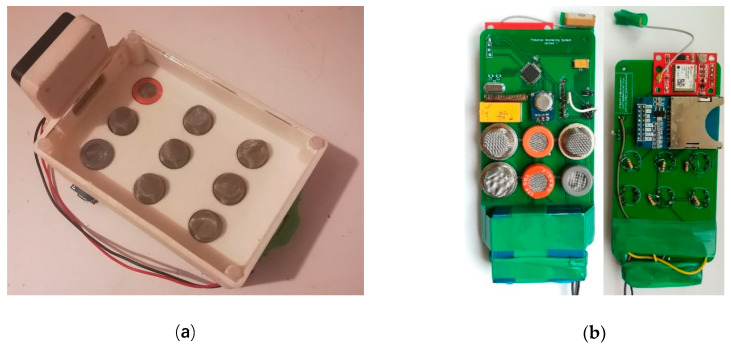
View of the assembled low-cost multi-sensor device in the stages of development: (**a**) prototype sensors, and (**b**) modified sensors.

The localization of the multisensory array is obtained from built-in NMEA protocol sentences [64] delivered by a GPS receiver on the universal asynchronous receiver-transmitter (UART) port and processed by the MCU. The localization can also be obtained by the drone’s onboard GPS, but the built-in system allows us to make 3D maps directly from sensors readings. The sampling rate is 1 Hz; every second, positional and pollution data are saved on the secure digital card(SD card) in comma-separated values file (CSV file) format. MCU is programmed to avoid file overwriting, and at every start-up it generates a new log file. At the same time, data are transmitted by the ultra high frequency (UHF) band (433 MHz). The transmitter uses a simple on/off keying (OOK) modulation. Small power of about 50 mW is enough to provide stable connection in the line of sight (LOS) flight mode. The data rate according to the STX882 transmitter manual can be in the range from 0.1 to 9.6 kbps. The data transmission has been set to 1.8 kbps, because smaller speed has better anti-interference parameters, and this speed is enough to fit all the data into a one-second interval. The transmitter works as a bridge between the computer/smartphone and the sensor module. It receives data from the system on the drone and retransmits this data over Bluetooth. This is useful for plotting data on screen in real-time. Also, it is possible to connect the receiver module directly to the PC using just a USB cable. The total cost of the developed multi-sensor device is below 70 EUR. Figure 4 shows the assembled low-cost multi-sensor module in the stages of development.

**Figure 5:**
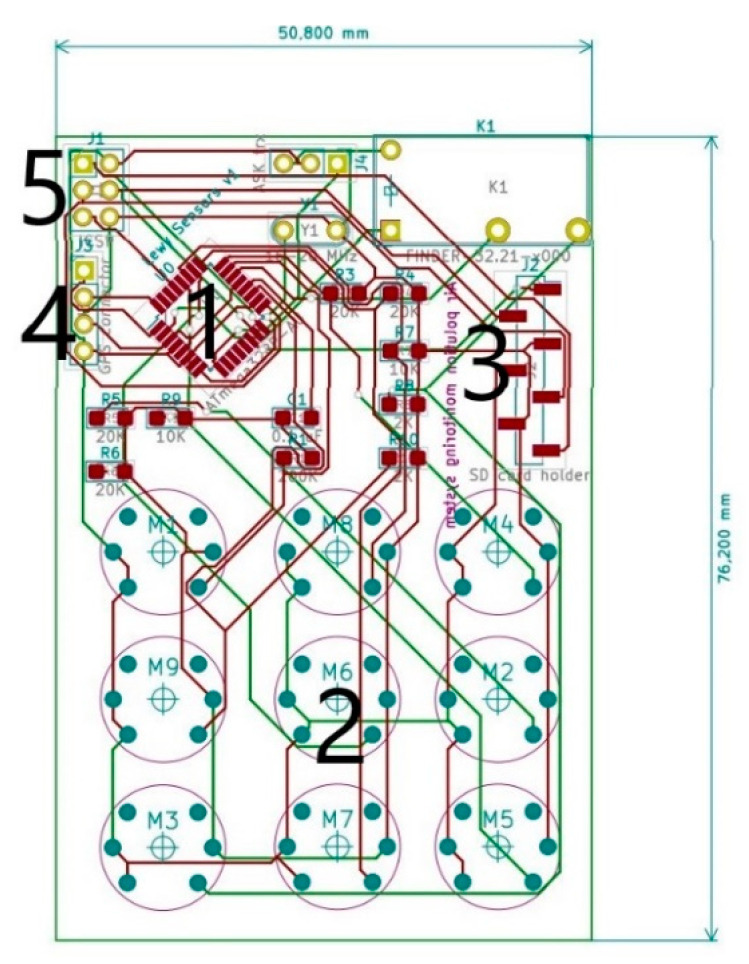
Schematic view of the first version of the integrated circuit board used for low-cost multisensory array assemble.

Figure 5 shows the original design scheme of the integrated circuit used for low-cost multisensory array assembly, and the measurements that are presented in this work. This was the very first prototype of the printed circuit board. This board had very simple construction and was based mostly on external modules like the external GPS module and the external SD card slot module. As labeled in Figure 5, on the board, the individual elements of the system are indicated as follows: 1—microcontroller unit (MCU), 2—air pollution sensors array, 3—SD card holder footprint, 4—external GPS device connector, and 5—programming connector. The board used in the research described in this paper was an improved version of one presented in Figure 5 and is described later on. The improvement concerned mostly the layout and, thus, the dimensions reduction.

**Figure 6:**
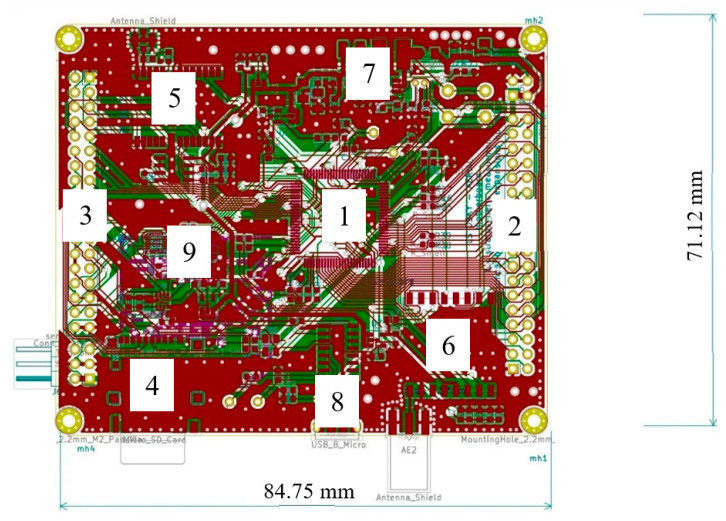
View of the modified version of the integrated circuit board.

For the prototypes, all of the software for microcontroller ATmega328 was written in Arduino IDE using the C++ programming language. Many versions of the dedicated software were created during the development of the project. The prototype was designed using sensors breakout boards, breadboards, and Arduino. After developing the software and testing the sensors, we decided to move the project onto a printed circuit board. The first version of PCB was very simple, and it had several mistakes, for example, the power traces that fed the sensors were too narrow. As a result, the sensors were not properly powered, and the signal trace was not surrounded by the ground zone. Generally, the system was not protected against interferences that were generated from the drone. The next version was designed to deal with previous problems, low pass filters were added to the project, which suppressed noise on analog lines that comes from the drone engines. Also, the range of the wireless communication was increased by switching to other microchips and changing the radio frequency (RF) modulation mode from Amplitude-Shift Keying (ASK), to Gaussian Frequency-Shift Keying (GFSK), and in the next version of the device to Long Range Wide Area Network (LoRaWAN). In the actual version, the navigation has been improved by using other sensors that are independent from the GPS system. The microcontroller was changed to more effective 32-bit STM32F103VET6 with a maximum core frequency of 72 MHz. Figure 6 shows a modified version, an integrated circuit, which has a connector for the quick exchange of single sensors during measurements. Such a solution allows us to reduce the dimensions of the device, which significantly reduces the weight of the whole chip, and this allows for the use of drones with a lower lifting force for the field measurements. Also, this version has an integrated barometer, gyro, and accelerometer, which are used for the inertial navigation. These features allow the device to take mobile measurements inside buildings or other places where GPS signal is not strong enough to provide stable three-dimensional position. The communication system is based on SX1276 from and by the user choice device, and could transmit data using LoRa or GFSK modulation. The device is compatible with LoRaWAN gateways, which makes this device universal and suitable for mobile measurements and for long range sensors networks as well. As labeled in Figure 6, the individual elements of the system are as follows: 1 is the MCU (processing unit), 2 and 3 are multipurpose connectors that can be used with additional sensor modules, 4 is the SD card holder, 5 is the high precision 10 Hz GPS with an external antenna connector, 6 is the long range communication system that uses GFSK modulation, 7 is the power module that is designed to provide stable voltage output at large temperature changes, 8 is the micro USB port that enables the device to be configured before flight, and 9 is the barometric altimeter and three axis accelerometer, gyroscope, and magnetometer that is used to detect the orientation of the device. 

The weight of the assembled device is limited because the drone can only lift a certain weight. Sensors are packed into a small chamber with fan forced circulation. The sensor’s case was created using 3D printing technology. A handy prototype frame was printed using lightweight Acrylonitrile butadiene styrene plastic (ABS plastic); its weight was 95 g. The weight of the measuring system was 108 g, while the separate power supply system was 363 g. Total mass of the device was 566 g, and it did not exceed the maximum lift-off weight of the UAV. 

**Figure 7:**
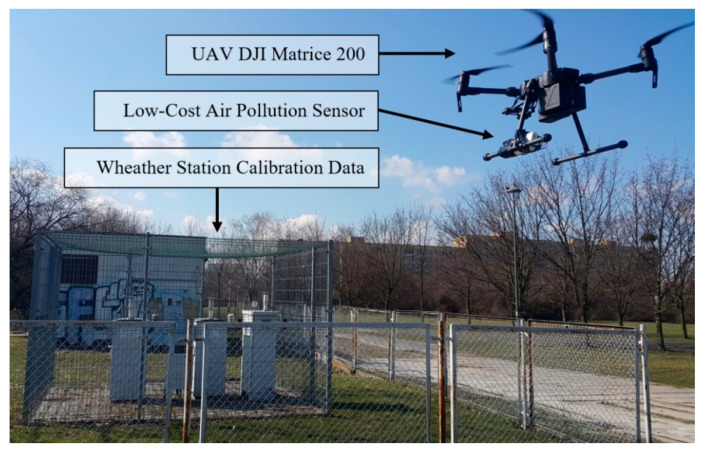
View of the mounted low-cost multi-sensor on the UAV during operation in location 2 (see Figure 1).

The flights were carried out by means of the DJI Matrice 200 drone. This UAV is designed for industrial applications. It has solutions that help to expand its measuring equipment according to the desired needs. The quadrocopter is equipped with DJI’s high performance 3515 model engines and 17-inch nylon propellers that allow it to fly during wind speed of up to 10 m/s. It also has a double battery charging system, which heats up automatically when the ambient temperature is below 0 ℃. Two types of DJI batteries were used for the test. The TB50 model has a capacity of 4280 mAh, and this allows it to fly up to 27 min, while the second TB55 battery has a capacity of 7660 mAh, and this allows it to fly up to 38 min at a flight velocity of up to 83 km/h. The drone’s construction meets the IP43 standard. It is able to fly in highly unfavorable environmental conditions such as heavy rain or heavy dust and in temperatures from −20 °C to +45 °C. The size of the unfolded unmanned aircraft is 887 mm long, 880 mm wide, and 378 mm high. It weighs from 3800 g to 4500 g, and can take a load of 1600 g to 2300 g depending on the batteries used. The quadrocopter has sensors installed at the front, top, and bottom to allow for planned flights and to assist the pilot during the flight. It can track an object and perform a flight around it while avoiding obstacles it encounters during the test. When the drone is performing the flight, it is possible to transmit a constant Lightbridge in 2.4 G Full HD quality. The Matrice 200 is also equipped with DJI AirSense, which transmits information about objects that are in the airspace. During the flight, it uses GPS and GLONASS networks. Figure 7 shows the view of the mounted low-cost multi-sensor on the UAV DJI Matrice 200 during operation in location number 2 (see Figure 1) on 5 March 2020. 

The battery capacity mainly determines the flight duration. The UAV range also depends on flight velocity and weather conditions. The basic limitation of the distance covered during a measurement flight is also the wireless communication range. In Europe, the communication range is limited to 2500 m. The flight altitude depends on a given aviation zone (CTA) and ranges from 0 to 2500 m. The airspace structure is built from the aviation zones. The nominal flight altitude outside of the controlled space is 2500 m. 

The measurements were taken in the following order: every 5 m of altitude, a 15 s hovering was performed and a measurement was taken. Therefore, the UAV movement velocity is not relevant in this case. For each location, five measurement flights were made in one day with stable weather conditions. The results were averaged for each altitude.

Measurements of the atmosphere condition with the application of unmanned aerial vehicles (UAV) is a forward-looking way to learn and then reduce the negative impact of human activity on the quality of the atmosphere. The designed air pollution multisensory array is an easy to assemble and user friendly extremely low-cost device (70 EUR) with high measurement accuracy, which will be shown in the results section. 

All compounds were measured; however, due to the limited space in the manuscript, selected results were included whose levels were significant for this specific location. The phenomenon of turbulence is an important issue that is expressed by the analyses of other authors [65,66]. On the day of the measurement, the weather conditions were stable with no wind. The turbulence that could have appeared in the drone’s propeller stream was not taken into account because the mounting of the sensor on the UAV was out of the propellers’ range and the design of the casing additionally protected the sensors against air turbulence. 

## 3. Results and Discussion

**Figure 8:**
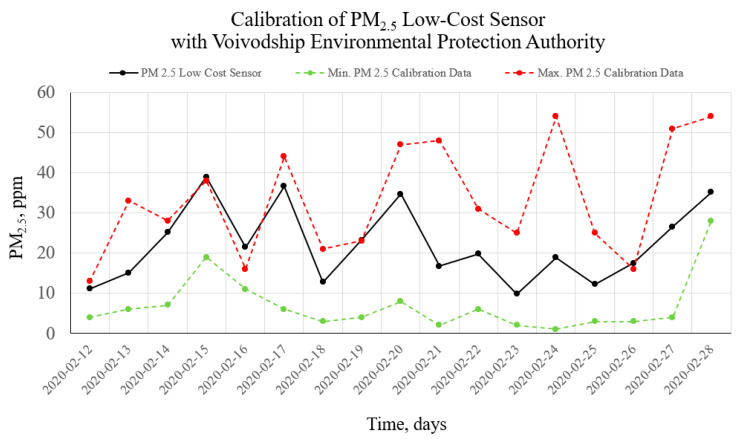
Calibration data from a two-week measurement period. Comparison of minimum (green) and maximum (red) readings from the reference pollution measurement station with the measurement results using the developed sensor (black).

As a result of the performed measurements, the particulate matter air pollution was measured in the specified areas, and vertical air pollution profiles were visualized. To make proper measurements, the sensors were calibrated. Calibration measurements were made during a two-week continuous period on 12–28 February 2020. The calibration was performed based on the comparison of minimum and maximum readings from the reference pollution measurement station (see Figure 7) with the measurement results from the developed sensor. The calibration station is part of the Polish national environmental monitoring system, whose measurement data are openly accessible [67]. The results of calibration measurements are shown in Figure 8. 

Figure 8 shows comparative graphs from the low-cost sensor readings on the background of the readings from the reference weather station. It can be seen that during the tested two-week period, the readings from the low-cost sensor are within the scatter of readings from accredited sensors in the reference weather and air pollution measuring station.

The sampling time for the low-cost setup is 15 s, while the reference station provides 15-min averages. Thus, to present the trend for the reference station in Figure 8, we averaged the measurement data. We did this to show the trend because in some cases cheap sensors may tend to overstate the measured values. This averaging is very important to check that a cheap laser sensor does not get dirty over time, which could lead to a deterioration of the measurements. More accurate calibration analyses are in line with other authors [68].

For future analyses of the impact of weather conditions on air pollution, the results of the measurements of the weather parameters such as outside temperature, outside humidity, wind speed, rain amount, and barometric pressure were also gathered. Long-term measurements are required to characterize the impact of weather conditions on the value of individual types of pollution in a uniform way. Table 2 shows the collected weather data from the local weather station for the sensor calibration period (the last row shows the condition during the test time).

**Figure 9:**
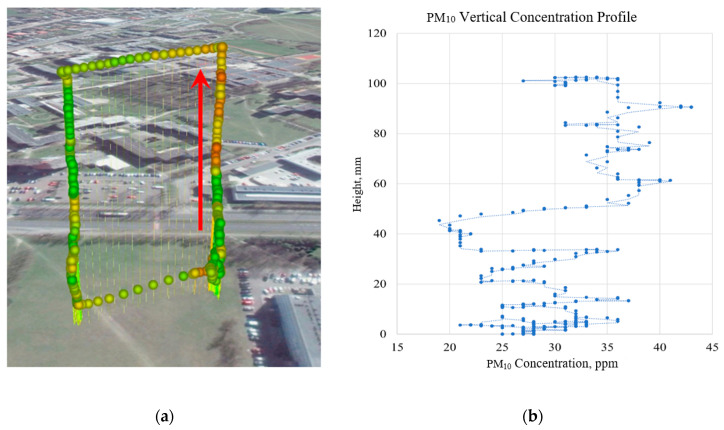
Flight route and measurement points visualization (**a**) for the vertical concentration profile of PM_10_ (**b**) for location number 1 (see Figure 1)—red arrow indicates ascending flight and data for the visualization chart.

**Figure 10:**
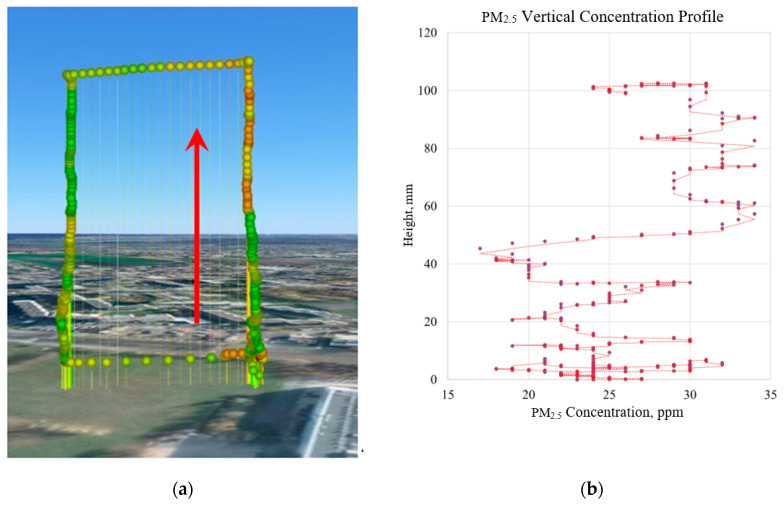
Flight route and measurement points visualization (**a**) for vertical concentration profile of PM_2.5_ (**b**) for location number 1 (see Figure 1)—red arrow indicates ascending flight and data for the visualization chart.

**Figure 11:**
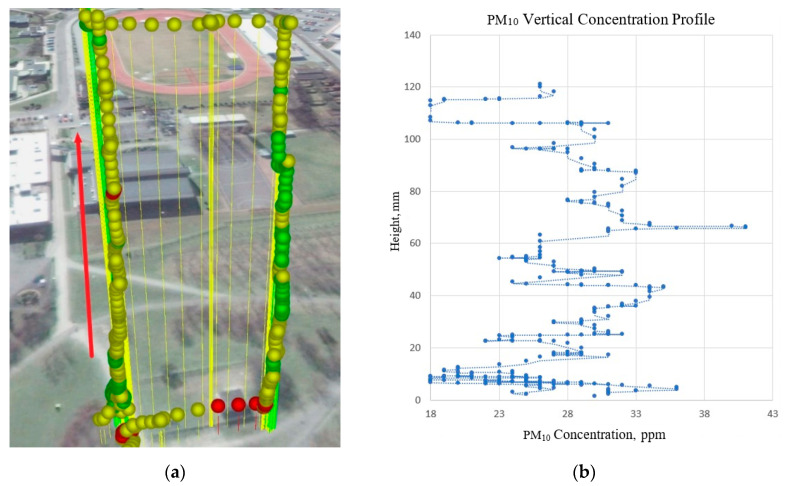
Flight route and measurement points visualization (**a**) for vertical concentration profile of PM_10_ (**b**) for location number 2 (see Figure 1)—red arrow indicates ascending flight and data for the visualization chart.

**Figure 12:**
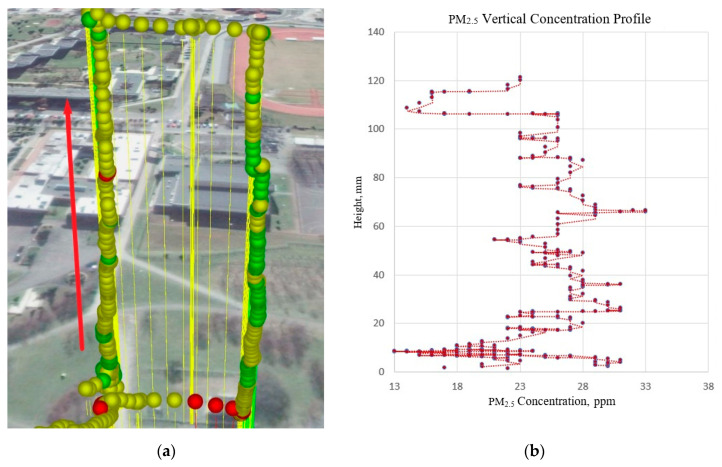
Flight route and measurement points visualization (**a**) for vertical concentration profile of PM_2.5_ (**b**) for location number 2 (see Figure 1)—red arrow indicates ascending flight and data for the visualization chart.

Measurements with the use of low-cost air pollution multi-sensor system mounted on UAV were made on 5 March 2020. Figure 9, Figure 10, Figure 11 and Figure 12 show examples of vertical concentration profiles for PM_2.5_ and PM_10_ for location number 1 and location number 2 (see Figure 1).

On the presented exemplary results of the air pollution in the form of PM concentration measurements in location number 1 (see Figure 1) located close to a busy road, it can be seen that even though there were relatively low concentrations in the ground layer, at heights from 60 m upwards, the concentrations of pollutants increased significantly. Descending flight shows lower values due to the influence of the propeller jet.

On the presented exemplary results of the air pollution in the form of PM concentration measurements in location number 2 (see Figure 1) located in a large urban recreational park at a considerable distance from busy roads (500 m), it can be seen that although there were relatively low concentrations in the ground layer, at heights from 60 m upwards, the concentrations of pollutants increased significantly up to about 100 m. Then, the pollution concentration rapidly decreases. In this case, the descending flight also shows lower values due to the influence of the propeller jet.

**Figure 13:**
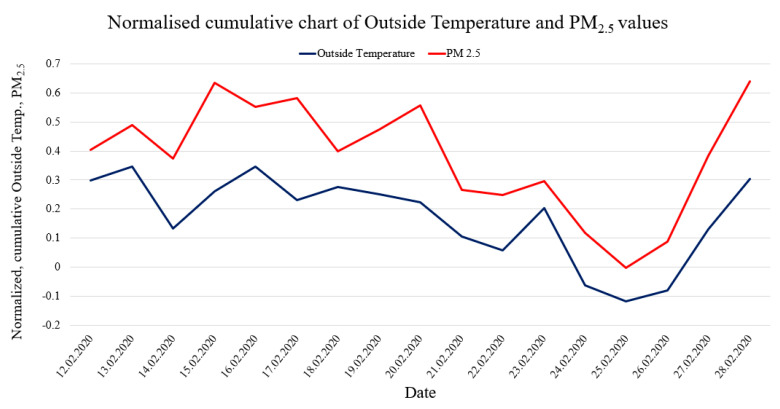
Comparison of standardized and cumulative values of the outside temperature and the PM_2.5_ concentration.

Another issue worth considering is the relationship between the properties of the atmosphere in its lower layers, such as temperature, humidity, precipitation, pressure, haze, etc., and the content of pollutants in the air. Figure 13 shows an example of such a comparison to search for correlations. To be able to present the quantities with different units in a single diagram, the values have been standardized.

Figure 13 clearly shows a correlation between the outside temperature and air pollution by particulate matter. Although the temperature increase in the long term should cause a decrease in PM emissions due to a reduction in household heating, the weather was extremely stable during the relatively short two-week period of time during which the sensors were calibrated, as shown in Table 2. In particular, the pressure and humidity were fairly constant, the wind speed was low, and there was no precipitation at all. This may also be the reason for the lifting of the dust that was near the ground surface to higher levels. Therefore, it seems that short-term correlation can occur under such stable conditions. This is a very interesting phenomenon that requires further observations.

## 4. Conclusions

The research shows that air pollution spreads unevenly across the Earth’s atmosphere depending on the many factors, such as height, season of the year, and time of the day. Weather conditions are also of significant importance, and should be monitored during the pollution measurements. The vertical profile of pollutant distribution should be monitored due to the possibility of accumulation in different layers of the atmosphere. Even though ABL stretches up to an altitude of about 2000 m, pollutants that may affect human health occur in significant exceedances mainly at altitudes not exceeding 400 m. Even if it may seem that it is too high for these pollutants to affect humans, the LIDAR studies showed that the atmosphere in the ABL undergoes intensive vertical mixing even above 500 m. The mixing effect occurs especially in the afternoon and evening hours, which can bring pollution to the lower layers of the atmosphere (closer to ground level). Moreover, the studies have shown that during the fog, the pollutants are concentrated at altitudes below 200 m. Therefore, it is very important to conduct UAV driven studies up to a height of about 200 m in a cyclic way to determine the behavior of pollutants in the higher layers of the atmosphere, which can have a significant impact on human health.

Examinations of the atmosphere’s condition with the application of unmanned aerial vehicles (UAVs, drones) is a progressive way to study the negative influence of human activity on the quality of the Earth’s atmosphere. This knowledge can be then used to take action to decrease these negative effects. A designed air pollution multisensory array is easy to build, user-responsive, and extremely low-cost device (70 EUR) with high measurement capability. The research has shown that cheap sensors can be a good source of information on the state of the atmosphere at various altitudes. This has been proven by comparing readings from low-cost sensors concerning accredited sensors from governmental air pollution monitoring. It has also been proven that low-cost sensors are insensitive to contamination and can operate over long periods without maintenance. An important conclusion of the conducted research is that cheap sensors should be compared with reference equivalents before measurements because cheap sensors tend to overstate the measured values. Thanks to the studies carried out at the tested location, it has been shown that the distribution of contaminants is variable with altitude, and the concentration of PM can increase with altitude from 0 to 100 m in the range of 30% to 50% of measured values, which, combined with the vertical movements of the atmosphere, can have a significant impact on humans.

Developed types of cheap, small, and lightweight devices can be successfully used for cyclic and frequent measurements by creating atmosphere quality surveys, especially in cities and heavily urbanized spaces. It is not difficult to imagine a few, a dozen, or even tens of such devices performing coordinated flights monitoring the condition of the atmosphere on a swarm scale. This is much more relevant than depending on one or two ground-based measuring points, which are currently used in cities in Poland. 

## Figures and Tables

**Table 1:** Low-cost sensors parameters used for the multisensory array design.

Sensor	Gas Types	Measurement Range	Data Output	Power Consumption	Resolution	Price
MQ-2	LPG, Propane, CH_4_	300–10,000 ppm	analog	<900 mW	due to adc = 10 bit	1€
MQ-3	Ethanol, Hexane, Benzine	0.05–10 mg/L	analog	<750 mW	due to adc = 10 bit	1€
MQ-4	CH_4_, LPG, H_2_	200–10,000 ppm	analog	<750 mW	due to adc = 10 bit	1€
MQ-5	iso-butane, propane	200–10,000 ppm	analog	<800 mW	due to adc = 10 bit	1€
MQ-6	iso-butane, propane	200–10,000 ppm	analog	<750 mW	due to adc = 10 bit	1€
MQ-7	CO, H_2_	20–2000 ppm	analog	<350 mW	due to adc = 10 bit	1€
MQ-8	H_2_	100–10,000 ppm	analog	<800 mW	due to adc = 10 bit	2€
MQ-135	C_6_H_6_, NH_3_	10–1000 ppm	analog	<800 mW	due to adc = 10 bit	1€
TGS 822	C_6_H_6_, Acetone, Hexane	50–1000 ppm	analog	<660 mW	due to adc = 10 bit	6€
PMS 7003	PM_1.0_, PM_2.5_, PM_10_	0–1000 μg/m^3^	digital	<500 mW	1 ug/m^3^	13€

**Table 2:** Weather data from a local meteorological station.

Date	Outside Temperature, °C	Outside Humidity, %	Wind Speed, m/s	Rain Amount, mm	Barometric Pressure, hPa
12 February 2020	3.11	82.99	0.41	0.00	1024.32
13 February 2020	3.61	84.34	0.39	0.00	1021.96
14 February 2020	1.39	84.51	1.26	0.00	1018.86
15 February 2020	2.73	77.65	1.79	0.00	1020.67
16 February 2020	3.61	83.55	0.16	0.00	1028.77
17 February 2020	2.42	86.26	0.93	0.00	1024.38
18 February 2020	2.89	87.79	0.38	0.02	1023.03
19 February 2020	2.62	90.55	0.32	0.01	1031.20
20 February 2020	2.34	85.43	0.47	0.00	1043.93
21 February 2020	1.11	84.23	0.72	0.00	1039.25
22 February 2020	0.61	87.36	2.25	0.00	1029.62
23 February 2020	2.11	83.91	1.01	0.00	1032.00
24 February 2020	−0.65	84.10	0.30	0.00	1022.98
25 February 2020	−1.24	85.48	0.22	0.00	1021.24
26 February 2020	−0.84	86.71	0.16	0.00	1019.42
27 February 2020	1.35	85.82	0.51	0.00	1013.97
28 February 2020	3.16	83.21	1.88	0.00	1000.50
05 March 2020	4.00	77.10	4.93	0.00	1004.75
